# Dynamic Modeling Using Artificial Neural Network of *Bacillus Velezensis* Broth Cross-Flow Microfiltration Enhanced by Air-Sparging and Turbulence Promoter

**DOI:** 10.3390/membranes10120372

**Published:** 2020-11-27

**Authors:** Aleksandar Jokić, Ivana Pajčin, Jovana Grahovac, Nataša Lukić, Bojana Ikonić, Nevenka Nikolić, Vanja Vlajkov

**Affiliations:** Faculty of Technology Novi Sad, University of Novi Sad, Bulevar cara Lazara 1, 21000 Novi Sad, Serbia; paj@tf.uns.ac.rs (I.P.); johana@uns.ac.rs (J.G.); nlukic@tf.uns.ac.rs (N.L.); prodanic@tf.uns.ac.rs (B.I.); frinensy@gmail.com (N.N.); vanja.vlajkov@uns.ac.rs (V.V.)

**Keywords:** microbial biomass, cultivation, membrane separation, permeate flux, Kenics static mixer, fouling mitigation, ANN, training algorithm, transfer function, architecture

## Abstract

Cross-flow microfiltration is a broadly accepted technique for separation of microbial biomass after the cultivation process. However, membrane fouling emerges as the main problem affecting permeate flux decline and separation process efficiency. Hydrodynamic methods, such as turbulence promoters and air sparging, were tested to improve permeate flux during microfiltration. In this study, a non-recurrent feed-forward artificial neural network (ANN) with one hidden layer was examined as a tool for microfiltration modeling using *Bacillus velezensis* cultivation broth as the feed mixture, while the Kenics static mixer and two-phase flow, as well as their combination, were used to improve permeate flux in microfiltration experiments. The results of this study have confirmed successful application of the ANN model for prediction of permeate flux during microfiltration of *Bacillus velezensis* cultivation broth with a coefficient of determination of 99.23% and absolute relative error less than 20% for over 95% of the predicted data. The optimal ANN topology was 5-13-1, trained by the Levenberg–Marquardt training algorithm and with hyperbolic sigmoid transfer function between the input and the hidden layer.

## 1. Introduction

*Bacillus velezensis*, a species of the genus *Bacillus* [1], has been extensively studied in recent years due to its remarkable biocontrol qualities against different plant pathogenic bacteria and fungi [2]. Besides an ability to produce large number of metabolites responsible for its antibacterial and antifungal activity, such as lipopeptides [3], enzymes [4] and volatile organic compounds [5], biomass of *Bacillus velezensis* could also be successfully utilized as a biocontrol agent [6,7]. Since multiplication of bacterial biomass is predominantly performed in a liquid culture, separation of bacterial biomass from the cultivation broth is of a great importance.

Membrane separation processes such as microfiltration are gaining interest as an alternative to conventional separation processes and recently it has become the mainstream separation techniques used for cell harvesting and broth clarification [8]. Sustainability issues dominating today’s industry have contributed to the shift from chemical synthesis processes to biotechnology production in numerous branches of industry, so the membrane-based separations have become very promising unit operations [9].

However, one of the foremost problems, limiting the industrial application of cross-flow microfiltration of biological suspensions, is membrane fouling. The main concern of such complex phenomena as membrane fouling is a decrease of permeate flow during the filtration operation. In fact, this leads to a reduction in system productivity and makes cleaning of the membrane necessary. Thus, increased costs and shortened life of a membrane operation are the consequences. Cross-flow microfiltration, with feed flow tangential to a membrane surface, is one of the methods used to increase permeate flux and reduce the necessity for frequent membrane cleaning [10]. Nevertheless, the problem of fouling is still a major issue that needs to be addressed.

The cultivation broth is a complex mixture of whole microbial cells or cell debris, as well as residual medium components and various extracellular macromolecules. The decreasing pattern of microfiltration permeate flux during cultivation broth filtration is caused by numerous factors that can be evaluated by hydraulic resistance [8,11]. The hydraulic resistance associated with the cake build-up is the main factor influencing the microfiltration of cultivation broths [8,11,12]. The reduction of cake layer, and consequently permeate flux improvement, can be achieved by applying flow alternations in a membrane channel [13,14,15,16,17].

There are quite a few hydrodynamic methods for permeate flux improvement, which are based on the creation of unstable, non-stationary and turbulent feed flow patterns through membrane modules. Different types of static or dynamic turbulence promoters cause radial mixing as well as an increase in tangential feed flow rate, leading to higher permeate flux values. Static turbulence promoters show several advantages compared to dynamic ones. The main advantages are simpler installation, lower investment cost, lower operational and maintenance cost, longer lifespan and possibility to be used in wide range of feed flow-rates and viscosities [18]. The Kenics static mixer has been successfully applied for permeate flux improvement in different microfiltration processes using yeast cell suspension [13,19], bentonite suspension [20] and wastewater treatment [21]. The use of two-phase flow (gas-sparging) is also used in order to create flow instabilities in a membrane channel [22,23,24]. Furthermore, combination of the Kenics static mixer and gas sparging has also given satisfying results in terms of permeate flux improvement i.e., fouling mitigation [25,26].

Modeling of permeate flux decline represents an intricate problem due to complexity of the phenomena occurring during the microfiltration processes [9,27]. It is usually based on theory (applying physical, chemical, and hydrodynamic modeling parameters) or on empirical correlations developed on specific experimental data [28,29]. Considering the wide range of different phenomena during fouling of microfiltration membranes, a development of a flexible model is very difficult, thus, the dynamic models of microfiltration are relatively rare [27]. Frequently, traditional models are inadequate when applied for complex biological and inorganic feeds [30]. Modeling of flux decline can be very challenging for microfiltration of complex mixtures (such as cultivation broths), especially in combination with complex flow arrangements related to the application of the techniques for fouling mitigation [31]. For that reason, application of so called black-box models (such as artificial neural networks, ANN), is much simpler compared to traditionally established models. ANN do not require detailed knowledge of the system, which is especially important when functional dependence between the system inputs and outputs has not been clearly formulated or defined [32].

ANN have been used as an useful tool for modeling and simulation of microfiltration in order to describe complex non-linear systems with large number of correlated parameters, which could not be described using simple mathematical equations [33,34]. One of the first dynamic neural network models for microfiltration was related to raw cane sugar juice microfiltration, which showed convergence of 97% with experimental results, indicating that a neural network model could predict hydrodynamic membrane resistance with high accuracy [35]. Hamachi et al. [36] have used a neural network model to investigate the effects of different operational conditions on permeate flux and filtration cake thickness during microfiltration of bentonite suspensions.

Chellam [37] has used neural networks for prediction of permeate flux value using several different suspensions within a wide range of hydrodynamic parameters (initial transmembrane pressure, initial permeate flux, shear rate, feed concentration). This research has revealed that one hidden layer was usually enough for accurate prediction of permeate flux, while an increase in the number of hidden neurons has led to overtraining of the neural network model. On the other hand, Aydiner et al. [38] have concluded that a neural network with too small number of neurons in the hidden layer will not approximate non-linear relations appropriately. A similar conclusion was drawn by Fu et al. [39], who have predicted adsorption of the bovine serum proteins into polyethylene membrane using ANN, indicating a necessity to appropriately define neural network architecture in order to use it as a reliable prediction tool. Furthermore, neural networks trained using the common Levenberg–Marquardt training algorithm, were successfully applied in modeling of microfiltration of different suspensions [34,40,41,42,43,44,45,46,47]. Bayesian regularization was used as a training algorithm for neural network prediction of permeates flux during microfiltration of starch industry wastewater [31]. Liu et al. [20] have developed a neural network model for permeate flux prediction during microfiltration of calcium carbonate suspensions with the application of turbulence promoter as a method for permeate flux improvement. Neural networks were also applied for modeling of microfiltration of red plum juice [48] and sugar beet juice suspension [49]. ANN applied in modeling of microfiltration processes have usually used tangential, sigmoid or logistic transfer functions. 

In contrast to the previous studies [15,31], the novelty of the approach adopted in this work is training of the single neural network that could be used as simulation tool for the different modes of microfiltration operation. The modes of the operation include the use of hydrodynamic methods (air-sparging and static mixer) for flux decline lessening. Considering the previous research in the field of ANN application for dynamic modeling of microfiltration [15,31], the aim of this study was to investigate the performance of non-recurrent feed-forward network with one hidden layer in prediction of permeate flux value during microfiltration of *Bacillus velezensis* cultivation broth. The main goal was determination of the optimal neural network architecture for dynamic permeate flux prediction under various conditions. The data for ANN modeling were collected by the microfiltration experiments in the system without hydrodynamic methods for permeate flux improvement and with application of the Kenics static mixer or two-phase flow as fouling mitigation methods. Also, the experimental results obtained for the combination of these two methods were included in the data sets used for selection of the optimal neural network topology. 

## 2. Materials and Methods 

### 2.1. Preparation of Bacillus Velezensis Cultivation Broth

*Bacillus velezensis* cultivation broth has been produced by cultivation of the producing microorganisms in the Woulff bottles (total volume 2 L, working volume was 2/3 of the total volume) using the cultivation medium which contained 10 g/L of glycerol, 3 g/L of yeast extract, 3 g/L of (NH_4_)_2_SO_4_, 1 g/L of K_2_HPO_4_ and 0.3 g/L of MgSO_4_∙7H_2_O. pH value of the cultivation medium was set to 7.0 ± 0.2 before sterilization by autoclaving (121 °C, 2.1 bar, 20 min). Inoculum was prepared using nutrient broth (HiMedia Laboratories, Karnataka, India) under the following conditions: temperature 28 °C, external mixing rate 150 rpm, spontaneous aeration, duration 48 h. Cultivation medium in the Woulff bottles was inoculated with 10% (*v*/*v*) of inoculum. Cultivation conditions were as follows: temperature 28 °C, external mixing rate 150 rpm, aeration rate 0.75 vvm (volume of air∙volume of liquid^−1^∙min^−1^), duration 96 h. The resulting cultivation broth was used as a feed mixture for the microfiltration experiments. 

### 2.2. Microfiltration Experiments

Microfiltration experiments were carried out using the single channel ceramic membrane (Tami Industries, Nyons, France). The pore diameter was 200 nm, while the length and the inner diameter of the membrane were 250 mm and 7 mm, respectively. The effective membrane length was 230 mm, with filtration area of 0.0043 m^2^. Apparatus used in microfiltration experiments was described by Jokić et al. [16]. Recirculation of retentate and permeate in microfiltration experiments had assured constant feed conditions. The temperature was adjusted to 25 °C. Permeate flux (*J_P_*, L∙m^−2^∙h^−1^) value was calculated using the following equation (Equation (1)):

(1) $$J_{P}=\frac{V_{P}}{A\cdot t}$$

where *t* (h) is the time required to collect certain volume of the permeate *V_P_* (10 mL), and *A* (m^2^) is the effective filtration area.

Filtration experiments were carried out in four varying modes. The first set of experiments was conducted by using plain membrane without application of any method for permeate flux improvement. In the second set, the static mixer was used as a turbulence promoter to enhance filtration flux. Air sparging was employed as a method for flux decline mitigation in the third set, while the fourth set of experiments was used to investigate the combination of static mixer and air sparging.

The turbulence promoter used in microfiltration experiments was Kenics static mixer, made of stainless steel, with diameter of 6 mm and length of 230 mm (corresponding to the active membrane length). In the experiments with air sparging air flow rate was set and maintained constant using the electronic flow rate regulator (EL FLOW^®^ F 201AV, Bronkhorst, Ruurlo, The Netherlands). Experimental variables and their values used in the Box–Behnken experimental plan (3^3^—three variables varied at three levels) for microfiltration experiments are listed in Table 1.

### 2.3. Data Compilation

For development of the ANN model for dynamic prediction of permeate flux behavior during microfiltration of *Bacillus velezensis* cultivation broth all experimental data were joint in one single dataset. The time-dependent permeate flux values comprised 1115 experimental data points for all microfiltration modes. Operational conditions including microfiltration time, superficial feed velocity, transmembrane pressure and superficial air velocity were taken as four of five input variables for the ANN model in this study (Table 1). Experiments with or without static mixer were conducted in the same operational parameters range, so to make a distinction in the compiled datasets an additional input (the presence of the Kenics static mixer) was selected. The mode of operation with the static mixer was designated the value 1, and in the case of microfiltration without the static mixer the value was 0 (Table 1). Furthermore, the differences between values of superficial feed velocity and superficial air velocity in the operation modes with and without static mixer arose from the reduced effective cross section of the membrane channel due to the presence of the static mixer. The normalization of data was performed using the procedure according to Equation (2) [50]:

(2) $$J_{normal}=\left(1-\Delta^{L}-\Delta^{U}\right)\cdot \frac{J_{P}-J_{min}}{J_{max}-J_{min}}+\Delta^{L}$$

where *J_normal_* and *J_P_* are normalized permeate flux value and measured permeate flux value, respectively, *J_max_* and *J_min_* are maximal and minimal value of permeate flux in the series of experimental data, respectively, and Δ*^U^* and Δ*^L^* are upper and lower values of normalization limit, respectively (0.01 for each limit).

### 2.4. Artificial Neural Network Modelling

In order to obtain single neural network for simulation of all experiments including methods for improvement of permeate flux (turbulence promoter, air sparging and combination of the turbulence promoter and air sparging) during microfiltration of *Bacillus velezensis* cultivation broth, the neural network with one hidden layer was used. In this study, a non-recurrent feed-forward artificial neural network is used, as this type of neural networks is used in numerous studies that deal with ANN microfiltration modeling [27]. Optimal neural network architecture with the best prediction performance was selected among the four models of neural network. The models have combined two types of training algorithm—the Levenberg–Marquardt algorithm (*trainlm*) and Bayesian regularization (*trainbr*)—with two types of transfer functions between the input and the hidden layer: sigmoid logistic (*logsig*) and sigmoid hyperbolic (*tansig*). In this way, four ANN types were investigated (Table 2). In all ANN models, the transfer function between the hidden and the output layer was linear function (*puerlin*) (Table 2).

The Levenberg–Marquardt training learning algorithm is based on an improved method of error back-propagation and it uses an early training stop criterion in order to increase efficacy and training speed of the neural network. Training stop criteria are usually maximal number of epochs or the moment when the error decrease under the acceptable limit. Early training stop happens if generalization ability is not being improved during the training, or the model mean square error (MSE) starts to increase.

Bayesian regularization represents a modification of the Levenberg–Marquardt training algorithm which contributes to solving the problem of lowered generalization ability due to neural network model overtraining (reduced model accuracy when presenting a new unseen set of the data). Iterative procedure contributes to MSE lowering, but also to the reduced number of parameters which should be adjusted to achieve the lowest possible MSE, therefore this training algorithm minimizes the number of synaptic weights which should be adjusted during the neural network training. Hence, the accuracy of the Bayesian regularization is around five times higher compared to the Levenberg–Marquardt early-stop training algorithm [51].

The main goal of optimal neural network architecture is to select a simpler neural network, i.e., a neural network with minimal number of neurons in the hidden layer, which simultaneously provides satisfactory predictability of the network.

The normalized dataset of microfiltration experimental results was randomly divided into three groups, using the *randperm* algorithm in Matlab software (R2015b, MathWorks, Natick, MA, USA). The data used for ANN training consisted of 70% of all data, whilst 15% of data were used for validation and the rest 15% for model testing.

The criteria for the end of ANN training were maximal number of epochs 1500, minimal MSE 0 or minimal performance gradient 1∙10^−10^. The MSE for each neural network model was calculated for increasing number of neurons in the hidden layer from one to 15 neurons using Equation (3):

(3) $$MSE=\frac{1}{n}\overset{n}{\underset{i=1}{\sum }}{\left(J_{exp,i}-J_{pred,i}\right)}^{2}$$

where *n* is the number of data, *J_exp,i_* is ith experimental flux value and *J_pred,i_* is ith flux value predicted by the neural network.

Another criterion for optimal network selection was coefficient of determination (R^2^), calculated by Equation (4). The neural networks were trained 30 times, and the average MSE and R^2^ were calculated to avoid probabilistic weight selection influence:

(4) $$R^{2}=1-\frac{\sum_{i=1}^{n}{\left(J_{exp,i}-J_{pred,i}\right)}^{2}}{\sum_{i=1}^{n}{\left(J_{exp,i}-J_{pred,avg}\right)}^{2}}$$

where *J_pred,avg_* is the average flux value predicted by the neural network.

Validation of the neural networks models was performed using the linear regression analysis - Pearson’s correlation coefficient (*r*). The neural network model shows good correlation, i.e., good prediction capability, if the absolute value of the Pearson’s coefficient, calculated by Equation (5), is |*r*| ≥ 0.8:

(5) $$r=\frac{\sum_{i=1}^{n}J_{exp,i}\cdot J_{pred,i}-n\cdot {\bar{J}}_{exp}\cdot {\bar{J}}_{pred}}{\sqrt{\sum_{i=1}^{n}J^{2}-n\cdot {\bar{J}}_{exp}^{2}}\cdot \sqrt{\sum_{i=1}^{n}J_{pred,i}^{2}-n\cdot {\bar{J}}_{pred}^{2}}}$$

where ${\bar{J}}_{exp}^{}$ is an arithmetic mean of the experimental flux values and ${\bar{J}}_{pred}$ is an arithmetic mean of the flux values predicted by the neural network.

Relative importance of the input variables effect to permeate flux, as the output variable, was calculated using Garson’s equation (Equation (6)):

(6) $$v=\frac{\sum_{j=1}^{n_{h}}\left[\left(i_{vj}/\sum_{k=1}^{n_{v}}i_{kj}\right)o_{j}\right]}{\sum_{i=1}^{n_{v}}\left[\sum_{j=1}^{n_{h}}\left(\left(i_{vj}/\sum_{k=1}^{n_{v}}i_{kj}\right)o_{j}\right)\right]}$$

where *n_v_* and *n_h_* are number of the neurons in the input and the hidden layer, respectively, *i_j_* is the absolute value of connection weights between the input and the hidden layer neurons, and *o_j_* is the absolute value of connection weights between the hidden and the output layer neurons.

## 3. Results and Discussion

### 3.1. Effect of Learning Algorithm, Transfer Function and Number of Hidden Layer Neurons

**Figure 1:**
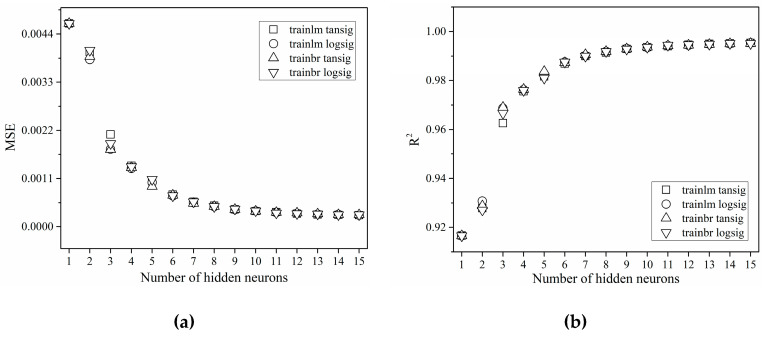
The effect of number of hidden neurons, training algorithm and transfer function between the input and the hidden layer to: (**a**) MSE (mean square error); (**b**) R^2^ (coefficient of determination).

A trial and error-based method was selected for defining the number of neurons in the hidden layer of the ANN. Figure 1 shows the variation of MSE and coefficient of determination versus the number of neurons in the hidden layer. The results are presented for the training set of data, and they clearly show that the outcome of increase in number of the hidden layer neurons is better predictability for all investigated networks. As can be seen, all network types investigated in this study had values of MSE and $R^{2}$ in a narrow range. This suggests that their predictive capacities were alike. Even for just a few hidden neurons high values of coefficients of determination were obtained.

In the case of the neural network model with the Levenberg–Marquardt algorithm and sigmoid hyperbolic function (network type B) minimal value of MSE was achieved using the neural network with 13 neurons in the hidden layer. Furthermore, the increase of the coefficient of determination could also be observed with the increase in number of neurons in the hidden layer. MSE and R^2^ values for this network were 2.69 × 10^−4^ and 0.99498, respectively. In case of using the Levenberg–Marquardt algorithm and sigmoid logistic function (network type A), minimum MSE value of 2.60 × 10^−4^ and maximum R^2^ value of 0.99539 were achieved for a neural network with 15 neurons in the hidden layer.

In the case of the neural network model with the Bayesian regularization and hyperbolic sigmoid function (network type D), a decrease of MSE could be also noticed when the number of neurons in the hidden layer was increased (Figure 1a). A minimal value of MSE and maximal value of R^2^ were achieved with 15 neurons in the hidden layer, 2.74 × 10^−4^ and 0.99513, respectively. On the other hand, when using neural network model with Bayesian regularization and hyperbolic logistic function (network type C), minimal value of MSE (2.74 × 10^−4^) and maximal value of R^2^ (0.99515) were achieved with 14 neurons in the hidden layer.

Therefore, it could be concluded that the optimal number of hidden neurons for approximation of the microfiltration results during microfiltration of *Bacillus velezensis* cultivation broth was 13. The neural network model trained by the Levenberg–Marquardt training algorithm and with hyperbolic sigmoid transfer function (network type B: *trainlm, tansig*) has shown the best prediction capability due to high values of coefficient of determination and MSE, with the lowest number of hidden neurons. Hence, the chosen optimal neural network was type B with architecture 5-13-1.

### 3.2. Verification of the Neural Network Model

**Figure 2:**
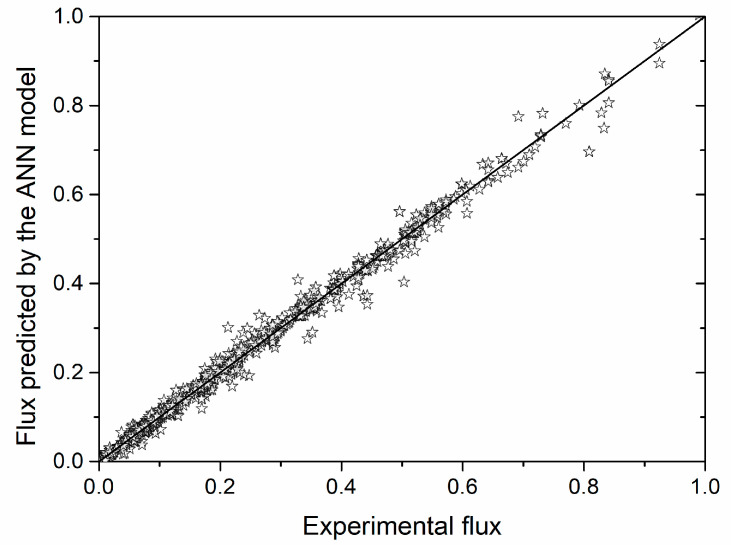
Normalized experimental permeate flux values plotted against the flux values predicted by the ANN (artificial neural network) model.

Prediction accuracy of the neural network model was checked using the Pearson’s coefficient (Equation (5)) and the coefficient of determination on experimental versus ANN data. Parity plot (Figure 2) for the complete dataset shows that there is a very close agreement between the experimental and the ANN predicted data for the great majority of cases. The Pearson’s coefficient value of 0.99611 suggested a good linear correlation between the experimental data and the data predicted by the neural network.

The coefficient of determination (R^2^) value of 0.99224 suggested that the linear regression equation for permeate flux could not explain less than 0.8% of the variations in the system. In other words, the majority of the data are close to the line which represents the ideal fitting of the experimental data (full line in the Figure 2, which represents ideal fitting by the linear model). This implies very good prediction consistency of the neural network model. Detailed estimation of neural network capability to predict permeate flux value during cross-flow microfiltration of *Bacillus velezensis* cultivation broth has been investigated using the analysis of absolute relative error (Table 3). The neural network model was able to predict 85% of the data with error less than 10%.

Furthermore, for 67% of the data the value of absolute relative error was less than 5%, while for only 6% of the data absolute relative error was higher than 20%. Considering that the neural network model for the experimental data for all microfiltration experiments has given predictions with absolute relative error less than 20% for 95% of the data, it could be concluded that ANN approach is suitable for prediction of permeate flux value during microfiltration of *Bacillus velezensis* cultivation broth.

**Figure 3:**
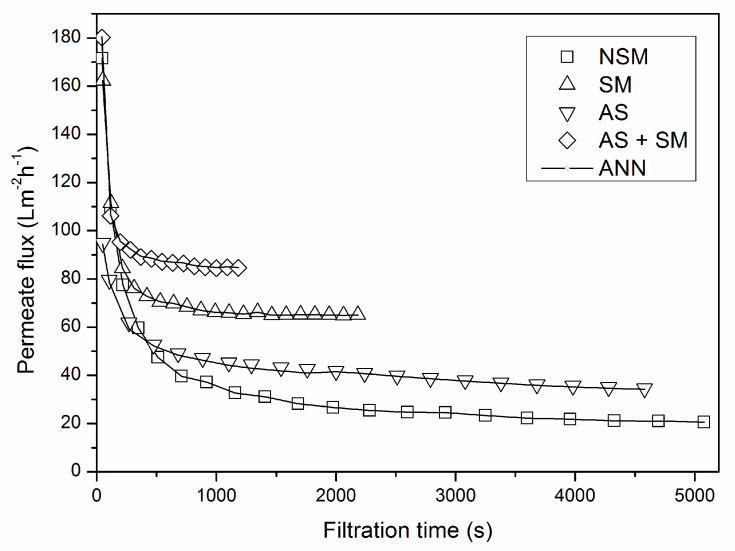
Prediction capability of the ANN model.

Additional microfiltration experiments were carried out at transmembrane pressure of 0.2 bar, superficial feed velocity of 0.43 m∙s^−1^ and for air-sparging experiments superficial air velocity was set at 0.2 m∙s^−1^. The experiments were undertaken with and without static mixer. The results of the simulation experiments are given in Figure 3.

In the initial microfiltration phase permeate flux value decline could be observed in all experimental setups. The reason for this can be found in the formation of a cake layer on the membrane surface. The layer made up of deposited microbial cells and large molecules causes an increase of the specific resistance to permeate flow, and thus the decrease in flux values. 

As expected, the lowest flux values were obtained without any fouling mitigation method used, i.e., in an empty membrane channel without static mixer (NSM mode). Air-sparging (AS mode) increased permeate flux values by around 100%, due to instabilities in feed flow caused by the existence of two-phase flow in the membrane channel. The presence of the Kenics turbulence promoter (SM mode) resulted in a significant increase of flux value (around 300%) for selected experimental conditions depicted in Figure 3. The characteristic shape of the mixer that intensifies radial mixing in the membrane channel and reduces the cake thickness is a probable reason for this [13,14,15]. Combination of the static mixer and two-phase flow (AS + SM mode) resulted in the highest flux values, although the contribution of air-sparging is less compared to the static mixer.

**Figure 4:**
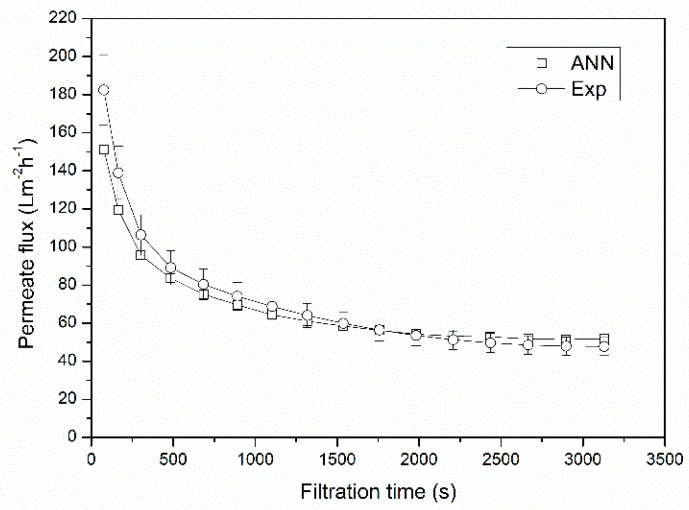
Generalization capacity of the ANN model.

Generalization capacity of the neural network model has been confirmed by a simulation of microfiltration experimental data, which had not been presented to the neural network in the phases of training, validation or testing. The experimental conditions of microfiltration variables in the presence of a turbulence promoter and with air-sparging (AS + SM mode) were set to 0.35, 0.6 and 0.3, for transmembrane pressure, superficial feed velocity and superficial air velocity, respectively. The results of the simulation experiment are given in Figure 4.

As can be seen in Figure 4, the selected ANN network is capable of satisfactorily simulating experimental data for the experiment. The majority of the predicted flux values falls in the range of 10% error. The most significant prediction error is noticed at the beginning of microfiltration operation. The reason for this can be found in the rapid flux decline at the beginning of *Bacillus velezensis* broth microfiltration. Cultivation broth is a complex mixture of various components that can cause severe membrane fouling in the initial phase of microfiltration [8,11,14,16]. This contributes to reduced ANN prediction capabilities in this region. A relatively small number of flux value experimental observations during the initial microfiltration period results in limited number of data provided for the network in the training stage. This limitation can be avoided by capturing more flux values in the initial microfiltration phase, thus improving predictability of the network at the beginning of the microfiltration period.

The results of a simulation of microfiltration experiments in all investigated modes indicate that a single neural network is capable of accurately predicting dynamic permeate flux values during microfiltration of *Bacillus velezensis* cultivation broth.

### 3.3. Relative Importance of the Input Variables

Although neural networks are considered to be black-box models, ignorant of real physical connections between experimental parameters, there are some possible ways to gain insights into the influence of experimental parameters on the microfiltration process. Considering weights and biases of the optimal trained neural network it is possible to assess the influence of the input parameters by the means of Garson equation (Equation (6)) [37]. Relative importance of the input variables is given in Table 4.

It could be concluded that filtration time has the most significant effect in determination of the permeate flux decline (50.30%). These results are in agreement with findings reported for dynamic modeling of *Streptomyces hygroscopicus* fermentation broth microfiltration [15], microfiltration of starch wastewater [31] and cross-flow microfiltration of a mixture that contains phosphate and fly ash [38].

The importance of superficial air and feed velocities were ranked second and fourth, respectively. The relative importance of superficial air velocity is around 4% higher compared to relative importance of superficial feed velocity, leading to the conclusion that increase of air velocity could contribute more to the change of permeate flux. Rod-shaped cells of *Bacillus velezensis* are oriented by the feed flow in the membrane channel [11,52]. As the feed flow velocity increases a turbulent flow regime is reached and influence of feed flow is less pronounced. On the other hand, when two-phase flow is applied, superficial velocities of the two-phase flow are increased and enhance turbulence due to movement of air bubbles, which change the cake layer structure. Consequently, superficial air velocity influences permeate flux more importantly compared to feed velocity.

The presence of the static mixer in the membrane channel is at the third place. Insertion of the turbulence promoter in the membrane channel significantly contributes to permeate flux increase, as shown in Figure 3. The Kenics static mixer causes the increase of feed velocity, hence the turbulence is caused by the unique flow pattern forming secondary flows and radial mixing which enable fluid movement closer to the membrane surface. This flow near the membrane surface increases permeate flux as it reduces the thickness of the cake layer and changes its brick like structure [16]. Furthermore, in the system with air sparging the presence of the turbulence promoter contributes to the significant change of a two-phase flow regimen by causing coalescence and bursting of the large air bubbles, as well as by making it possible for smaller air bubbles to flow near the membrane surface affecting breaking of the filtration cake structure, which lowers the permeate flow resistance leading to higher values of permeate flux and the steady state being reached faster.

The relative influence of transmembrane pressure has the lowest rank. It can be explained by the formation of a brick-like cake layer of the rod-shaped microbial cells. This type of cake structure becomes more compact with the increase of pressure, which in turn results in lower influence of transmembrane pressure on the permeation flux [11,14,52].

## 4. Conclusions

Considering the results presented in this study, it could be concluded that a single non-recurrent feed-forward ANN with architecture 5-13-1, trained by the Levenberg–Marquardt training algorithm with hyperbolic sigmoid transfer function between the input and the hidden layer, could be used as a reliable tool for modeling of permeate flux during microfiltration of *Bacillus velezensis* cultivation broth in various modes. The modes of the operation include the use of hydrodynamic methods (air-sparging and static mixer) for flux decline reduction. The proposed neural network model has predicted permeate flux values for all modes of microfiltration experiment. The accuracy of 99.23%, indicated by the coefficient of determination, shows absolute relative error less than 20% for over 95% of the predicted data. Furthermore, an analysis of relative importance of the input variables (microfiltration parameters) to permeate flux has revealed that the most noticeable effect has filtration time, followed by air linear velocity, presence of the Kenics static mixer, feed linear velocity and transmembrane pressure. The results of this study have confirmed a suitability of ANNs as a modeling tool for microfiltration, with an emphasis on the importance of adequate experimental data preparation, network architecture optimization, as well as on defining of appropriate neural network training parameters in order to obtain a highly accurate and reliable model.

## Figures and Tables

**Table 1:** Microfiltration experiments—input variables and their values.

Input Variable	Value	
	Without Static Mixer	With Static Mixer
Static mixer (-)	0	1
Transmembrane pressure (bar)	0.2; 0.6; 1.0	0.2; 0.6; 1.0
Superficial feed velocity (m∙s^−1^)	0.43; 0.87; 1.30	0.53; 1.06; 1.59
Superficial air velocity (m∙s^−1^)	0.0; 0.2; 0.4	0.0; 0.23; 0.46
Filtration time (s)	0—time to reach stationary flux	

**Table 2:** Neural network models investigated for microfiltration modeling.

ANN Type	Training Algorithm	Transfer Function	
		Input-Hidden Layer	Hidden-Output Layer
A	trainlm	logsig	puerlin
B	trainlm	tansig	
C	trainbr	logsig	
D	trainbr	tansig	

**Table 3:** Distribution of the absolute relative error of the neural network model.

Absolute Relative Error (%)	<1	<5	<10	<20	>20	Sum
Number of data	274	470	199	108	64	1115
Percentage of data (%)	25	42	18	10	6	100

**Table 4:** Relative importance of the input variables for *Bacillus velezensis* broth microfiltration.

Input	Importance (%)	Rank
Static mixer (-)	13.13	3
Transmembrane pressure (bar)	9.44	5
Superficial air velocity (m∙s^−1^)	15.77	2
Superficial feed velocity (m∙s^−1^)	11.36	4
Filtration time (s)	50.30	1
TOTAL:	100	
